# Характеристика гериатрического и соматического статуса у пациентов с остеопорозом

**DOI:** 10.14341/probl12751

**Published:** 2021-06-17

**Authors:** Н. О. Ховасова, А. В. Наумов, О. Н. Ткачева, Е. Н. Дудинская

**Affiliations:** Кафедра болезней старения Российского национального исследовательского медицинского университета им. Н.И. Пирогова; Российский геронтологический научно-клинический центр; Кафедра болезней старения Российского национального исследовательского медицинского университета им. Н.И. Пирогова; Российский геронтологический научно-клинический центр; Кафедра болезней старения Российского национального исследовательского медицинского университета им. Н.И. Пирогова; Российский геронтологический научно-клинический центр; Кафедра болезней старения Российского национального исследовательского медицинского университета им. Н.И. Пирогова; Российский геронтологический научно-клинический центр

**Keywords:** остеопороз, падения, переломы, гериатрический синдром

## Abstract

**ОБОСНОВАНИЕ:**

ОБОСНОВАНИЕ. Пожилые люди с остеопорозом (ОП) и высоким риском падений — наиболее уязвимая группа пациентов в отношении развития переломов. Падения и переломы у пожилых пациентов с ОП ассоциированы с гериатрическими синдромами и худшим функциональным статусом.

**ЦЕЛЬ:**

ЦЕЛЬ. Оценить коморбидный и гериатрический статус пациентов пожилого и старческого возраста с ОП и без него.

**МАТЕРИАЛЫ И МЕТОДЫ:**

МАТЕРИАЛЫ И МЕТОДЫ. В исследование включены 607 пациентов старше 60 лет, госпитализированных в гериатрическое отделение. По наличию ОП пациенты были разделены на 2 группы: 1 группа — пациенты с ОП (n=178, 29,3%), 2 группа — пациенты без ОП (n=429, 70,7%). Всем пациентам проводились общеклиническое исследование, оценка коморбидности по индексу Чарльсон, комплексная гериатрическая оценка.

**РЕЗУЛЬТАТЫ:**

РЕЗУЛЬТАТЫ. ОП имели 178 (29,3%) пациентов, чаще это были женщины. 55,6% пациентов с ОП были инвалидами. У пациентов с ОП достоверно чаще встречались возраст-ассоциированные заболевания — болезнь Альцгеймера, болезнь Паркинсона, остеоартрит, анемия, заболевания щитовидной железы, варикозная болезнь. Практически со всеми этими заболеваниями при однофакторном анализе выявлена ассоциация с ОП. У пациентов 1 группы достоверно чаще встречались такие гериатрические синдромы, как старческая астения, гиподинамия, недостаточность питания, полипрагмазия, недержание мочи. Пациенты с ОП чаще проживали одиноко и использовали вспомогательные средства при передвижении в сравнении с пациентами без ОП. Проведенный однофакторный анализ продемонстрировал, что ОП ассоциирован (отношение шансов (ОШ) 1,54–2,00) со старческой астенией, гиподинамией, использованием вспомогательных средств при передвижении, нарушением сна, сенсорным дефицитом по зрению, недержанием мочи. Функциональный статус пациентов с ОП был хуже по сравнению с пациентами без ОП. Пациенты с ОП перенесли больше переломов.

**ЗАКЛЮЧЕНИЕ:**

ЗАКЛЮЧЕНИЕ. Пациенты с ОП имеют высокую коморбидность, отягощенный гериатрический статус. У пациентов пожилого и старческого возраста необходимо не только проводить скрининг и диагностику ОП, оценивать риск 10-летней вероятности основных патологических переломов по алгоритму FRAX, но и проводить комплексную гериатрическую оценку для диагностики гериатрических синдромов, которые утяжеляют течение ОП и приводят к более серьезным последствиям.

## Abstract (en)

**BACKGROUND:**

BACKGROUND: Older adults with osteoporosis (OP) and high risk of falls are the most vulnerable group of patients with respect to the development of fractures. Falls and fractures in elderly patients with OP are associated with geriatric syndromes and worse functional status.

**AIM:**

AIM: To аssess comorbidity and geriatric status in elderly and senile patients with and without OP.

**MATERIALS AND METHODS:**

MATERIALS AND METHODS: The study included 607 patients over 60 years of age hospitalized in the geriatric department. According to the presence of OP, the patients were divided into 2 groups: group 1 — patients with OP (n=178, 29.3%), group 2 — patients without OP (n=429, 70.7%). All patients underwent a general clinical study, an assessment of comorbidity ­according to the Charlson index, and a comprehensive geriatric score.

**RESULTS:**

RESULTS: OPs had 178 (29.3%) patients, more often these were women. 55.6% of patients with OP were disabled. Age-­related diseases such as Alzheimer’s disease, Parkinson’s disease, osteoarthritis, anemia, thyroid disease, varicose veins were significantly more common in patients with OP. With almost all of these diseases, a univariate analysis revealed an association with OP. Geriatric syndromes such as frailty, hypodynamia, malnutrition, polypharmacy, urinary incontinence were significantly more common in group 1 patients. Patients with OP were more likely to live alone and use mobility aids compared to patients without OP.

The univariate analysis demonstrated that OP is associated (OR 1.54 to 2.00) with frailty, hypodynamia, the use of aids in movement, sleep disorders, sensory vision deficiency, urinary incontinence. The Functional status of patients with OP was worse compared to patients without OP. Patients with OP suffered more fractures, and vertebral fractures were significantly more frequent.

**CONCLUSION:**

CONCLUSION: Patients with OP have a high comorbidity, a burdened geriatric status. In elderly patients, it is necessary not only to screen and diagnose OP, to assess the risk of 10-years probability of major pathological fractures using the FRAX algorithm, but also to conduct a comprehensive geriatric assessment to diagnose geriatric syndromes that weaken the course of OP and lead to more serious consequences.

## ОБОСНОВАНИЕ

Остеопороз (ОП) — системное заболевание костной ткани, характеризующееся снижением костной массы и нарушением микроархитектоники кости [1]. Диагностические критерии ОП четко определены.

1. Наличие патологических переломов крупных костей скелета в анамнезе или выявленных при обследовании, независимо от результатов рентгеноденситометрии или FRAX.

2. Наличие высокой индивидуальной 10-летней вероятности основных патологических переломов независимо от показателя денситометрии.

3. Снижение минеральной плотности кости (МПК) на 2,5 и более стандартных отклонений по Т-критерию в шейке бедренной кости, и/или в целом в проксимальном отделе бедренной кости, и/или в поясничных позвонках, измеренной двухэнергетической рентгеноденситометрией, у женщин в постменопаузе и мужчин старше 50 лет [2].

ОП и остеопоротические переломы длительное время ассоциировались с женщинами в постменопаузальном периоде. Однако в современной реальности частота переломов растет из-за старения населения. В США сегодня ОП встречается у 46,3% мужчин и 77,1% женщин пожилого и старческого возраста [3]. В странах Западной Европы риск любого остеопоротического перелома в течение жизни составляет 40–50% для женщин и 13–22% для мужчин [4].

К 2050 г. в мире прогнозируется увеличение лиц пожилого и старческого возраста примерно в 2 раза. Однако увеличение продолжительности жизни не равно увеличению продолжительности здоровой и качественной жизни. Добавленные к жизни годы зачастую характеризуются заболеваниями и инвалидностью [5]. Согласно докладу Международного фонда по ОП, к 2040 г. число пациентов с ОП в возрасте старше 50 лет удвоится по сравнению с показателями 2010 г. [6]. В связи с этим вмешательства по снижению частоты новых случаев, а также прогрессирования уже имеющегося ОП и предотвращения переломов должны быть сосредоточены на пожилой категории населения [7].

Тяжелые медицинские, социальные и экономические последствия при ОП связаны прежде всего с низкоэнергетическими переломами, приводящими к хронической боли, депрессии, потере автономности, инвалидизации и смерти. Повышенная смертность после перелома проксимального отдела бедра в значительной степени зависит от возраста и выше у мужчин. Но в популяционном масштабе важно и то, что статистически значимая повышенная смертность сохраняется в течение 10 лет после случившегося перелома шейки бедра [8].

Одним из провоцирующих факторов переломов являются падения. Центры по контролю и профилактике заболеваний в США сообщают, что в 2014 г. почти 30% пожилых людей сообщили о падении. Возраст сам по себе является фактором риска падений: частота падений в 90 лет в 2 раза выше, чем в 60 лет [9]. Другие признанные факторы риска падений — это остеоартрит, депрессия, сердечно-сосудистые заболевания и недержание мочи [10].

Падения у пожилых пациентов с ОП, имеющих более высокий риск переломов, приводят к нарастанию нуждаемости в посторонней помощи, увеличению частоты госпитализаций и инвалидизации [11]. Поэтому пациенты с низкой МПК и высоким риском падений — наиболее уязвимая группа пожилых людей в отношении развития переломов. Риск падений и переломов у пожилых пациентов с ОП ассоциирован с гериатрическими синдромами — саркопенией, полиморбидностью и старческой астенией (СА) [12].

СА и ОП имеют общие факторы риска — возраст, низкая физическая активность, потеря массы тела, когнитивные нарушения. И до сих пор однозначно неясно, что первично — ОП или СА [13]. Ряд исследований демонстрирует связь тяжести ОП и риска переломов у пациентов со СА. Так, у женщин с синдромом СА риск переломов шейки бедра (отношение шансов (ОШ) 1,40; 95% доверительный интервал (ДИ) 1,03–1,90) более высокий, чем у крепких женщин того же возраста [14]. С другой стороны, СА имеет более тяжелое течение у женщин, которые перенесли остеопоротический перелом, что свидетельствует о большем накоплении дефицитов и ускорении прогрессирования СА после остеопоротического перелома [15]. В ряде исследований было установлено, что комплексная оценка по диагностике СА сопоставима с FRAX при прогнозировании риска основных остеопоротических переломов и переломов проксимального отдела бедра, что указывает на то, что оценка наличия СА может определять риск переломов у пожилых людей. В связи с чем сегодня обсуждается возможность интеграции параметров, характеризующих СА, в шкалу FRAX для более точного прогнозирования риска переломов [16].

Ряд исследований показывает связь между низкой МПК и саркопенией как у мужчин, так и у женщин пожилого возраста [17–19]. Locquet и соавт. показали на 232 пациентах старше 75 лет, что снижение мышечной функции связано с изменением микроархитектоники костей, а пациенты с саркопенией имеют 5-кратный повышенный риск развития ОП [20]. Сочетание саркопении и ОП (остеосаркопения) еще более повышает риск падений (ОШ 1,41; 95% ДИ 1,02–1,95) и остеопоротических переломов (ОШ 1,87; 95% ДИ 1,07–3,26) по сравнению с пациентами, не имеющими ни ОП, ни саркопении [21–23]. Кости и мышцы тесно взаимодействуют друг с другом не только анатомически, но и метаболически. Например, имеется перекрестная связь «мышца-кость» за счет факторов, синтезируемых мышцей, — инсулиноподобного фактора роста-1, фактора роста фибробластов, ИЛ-6, ИЛ-15, миостатина, остеоактивина, которые в том числе влияют на моделирование кости [24] и прогрессирование ОП [25]. Также нельзя забывать о значении хронического воспаления, являющегося следствием совокупного действия антигенной нагрузки в течение всей жизни, вызванной как клиническими, так и субклиническими инфекциями, а также воздействием неинфекционных антигенов. Следствием этого являются воспалительный ответ, повреждение тканей, высвобождение дополнительных цитокинов, что определяет порочный круг, приводящий к хроническому провоспалительному состоянию — inflammaging [26]. Системное воспаление, саркопения, ОП приводят к нездоровому старению, характеризующемуся развитием и прогрессированием гериатрических синдромов. С этой точки зрения представляется интересным охарактеризовать гериатрический портрет пациента с ОП.

## ЦЕЛЬ ИССЛЕДОВАНИЯ

Оценить коморбидный и гериатрический статус пациентов пожилого и старческого возраста с ОП и без него.

## МАТЕРИАЛЫ И МЕТОДЫ

Место и время проведения исследования

Исследование проводилось на базе ОСП «Российский геронтологический научно-клинический центр» ФГАОУ ВО РНИМУ им. Н.И. Пирогова Минздрава России, Москва, в период июнь 2019–февраль 2021 г.

Изучаемые популяции

Пациенты (n=607) старше 60 лет, госпитализированные в гериатрическое отделение. Критерии включения: возраст 60 лет и старше; согласие на участие в исследовании. Критерии исключения: возраст менее 60 лет; онкологические заболевания; тяжелая почечная недостаточность; тяжелая печеночная недостаточность; отказ от участия в исследовании.

Способ формирования выборки из изучаемой популяции: сплошной.

Дизайн исследования

Одноцентровое одномоментное наблюдательное исследование. По наличию ОП пациенты были разделены на 2 группы: 1 группа — пациенты с ОП (критерии: наличие патологических переломов костей скелета в анамнезе или выявленных при обследовании; высокая (для лиц 60–89 лет — более 20,12%, а для лиц 90 — более 18,8%) индивидуальная 10-летняя вероятность основных патологических переломов по алгоритму FRAX; снижение МПК на 2,5 стандартных отклонения и более по Т-критерию в шейке бедренной кости и/или в целом в проксимальном отделе бедренной кости и/или в поясничных позвонках), 2 группа — пациенты без ОП. Общая характеристика пациентов представлена в табл. 1.

**Table:** Таблица 1. Общая характеристика пациентов

	С ОП	%	Без ОП	%
Количество	178	29,3	429	70,7
Возраст	77,1±7,39		74,3±8,01	
Женщины	159	89,3	334	77,9*
Мужчины	19	10,7	95	22,1*
Инвалидность	99	55,6	164	37,4*

Методы

ОП диагностировался согласно диагностическим критериям: 1) наличие низкоэнергетических переломов крупных костей скелета в анамнезе или выявленных при обследовании, независимо от результатов рентгеноденситометрии или FRAX; 2) наличие высокой индивидуальной 10-летней вероятности основных низкоэнергетических переломов независимо от показателя денситометрии; 3) снижение МПК на 2,5 стандартных отклонения и более по Т-критерию в шейке бедренной кости, и/или в целом в проксимальном отделе бедренной кости, и/или в поясничных позвонках, измеренной двухэнергетической рентгеноденситометрией, у женщин в постменопаузе и мужчин старше 50 лет [2]. Для оценки коморбидного статуса всем пациентам было проведено общеклиническое исследование, рассчитан индекс коморбидности Чарльсон. Для характеристики гериатрического статуса всем пациентам проведена комплексная гериатрическая оценка (КГО) согласно методологии, описанной в российских клинических рекомендациях «Старческая астения» [27]. Оценку МПК проводили методом двухэнергетической рентгеновской абсорбциометрии с помощью аппарата Prodigy, GE Lunar (DXA). Измерялись суммарная МПК поясничного отдела позвоночника (LI-LIV), МПК области шейки бедренной кости, суммарная МПК бедренной кости. Денситометрическое исследование проводилось одним специалистом.

Статистический анализ

База данных создана в программе IBM® SPSS® Statistics version 23.0 (SPSS Inc., США). Вид распределения количественных переменных анализировали при помощи одновыборочного критерия Колмогорова–Смирнова. Количественные показатели приведены в виде среднего арифметического (М) с соответствующим стандартным отклонением (SD). Качественные данные представлены в виде абсолютных чисел и относительных частот. Для ненормально распределенных показателей применялся непараметрический критерий Манна–Уитни, для нормально распределенных — T-критерий Стьюдента. Взаимосвязи между переменными оценивали при помощи однофакторного анализа, для чего использовали бинарную логистическую регрессию с вычислением отношения шансов (ОШ) и 95% доверительного интервала (ДИ). Корреляционный анализ проводили с использованием ранговой корреляции Спирмена. Различия считали значимыми при р<0,05.

Этическая экспертиза

Протокол исследования рассмотрен и одобрен независимым этическим комитетом ОСП Российский геронтологический научно-клинический центр ФГАОУ ВО РНИМУ им. Н.И. Пирогова (протокол заседания № 25 от 17.06.2019).

## РЕЗУЛЬТАТЫ

Каждый третий пациент (29,3%) старше 60 лет страдал ОП, чаще это были женщины. У 96 (53,9%) человек ОП был подтвержден данными денситометрии, у 40 (22,5%) — высокой индивидуальной 10-летней вероятностью основных низкоэнергетических переломов, у 75 (42,1%) — наличием низкоэнергетических переломов костей скелета в анамнезе или выявленных при рентгенографии грудного и поясничного отделов позвонков, выполненной в двух проекциях. Необходимо отметить, что у 10 пациентов при наличии низкоэнергетических переломов в анамнезе на амбулаторном этапе диагноз ОП не выставлялся и не проводилось никакой антиостеопоротической терапии.

55,6% пациентов с ОП имели инвалидность, что в 1,5 раза чаще, чем у пациентов без него (табл. 1). Это, в том числе, можно объяснить более высокой распространенностью коморбидности у пациентов с ОП (табл. 2).

**Table:** Таблица 2. Распространенность и структура коморбидной патологии

	С ОП (n=178)	%	Без ОП (n=429)	%	р
Индекс коморбидности Чарльсон	6,04±1,72		5,52±2,02		<0,001
АГ	172	96,6	413	96,2	0,414
ИБС	84	47,2	195	45,5	0,343
ФП	33	18,5	84	19,6	0,383
ХСН	62	34,8	135	31,5	0,210
ОНМК, ТИА	26	14,6	58	13,5	0,442
СД 2 типа	45	25,3	107	24,9	0,462
Заболевания щитовидной железы	67	37,6	112	26,1	0,002
Ожирение	76	44,7	199	46,4	0,212
Болезнь Альцгеймера	4	2,2	2	0,5	0,021
Болезнь Паркинсона	12	5,6	10	2,3	0,002
Анемия	34	19,1	51	11,9	0,019
ХОБЛ	27	15,2	59	13,8	0392
Мочекаменная болезнь	28	15,7	64	14,9	0,244
Язвенная болезнь желудка и двенадцатиперстной кишки	34	19,1	73	17	0,275
Варикозная болезнь	86	48,3	170	39,6	0,024
Остеоартрит	144	80,9	220	51,3	0,005
Подагра	9	5,3	20	4,7	0,418
Доброкачественная гиперплазия предстательной железы	13	68,4	49	51,6	0,096

Полученные данные демонстрируют, что у пациентов 1 группы достоверно чаще встречались заболевания щитовидной железы, болезнь Альцгеймера, болезнь Паркинсона, анемия, остеоартрит, варикозная болезнь. Индекс коморбидности Чарльсон был выше также в 1 группе наблюдения. Далее при помощи однофакторного анализа была изучена взаимосвязь между ОП и соматическими заболеваниями. Выявлены ассоциации ОП с заболеваниями щитовидной железы, остеоартритом, анемией, варикозной болезнью вен и болезнью Паркинсона. С другими заболеваниями достоверной ассоциации выявлено не было (табл. 3).

При проведении комплексной гериатрической оценки были диагностированы различные гериатрические синдромы, структура которых представлена в табл. 4.

**Table:** Таблица 3. Ассоциации между остеопорозом и коморбидными заболеваниями

	ОШ	95% ДИ	р
Остеоартрит	4,02	2,65–6,11	<0,05
Болезнь Паркинсона	3,03	1,28–7,16	<0,05
Заболевания щитовидной железы	1,71	1,18–2,48	<0,05
Анемия	1,71	1,06–2,74	<0,05
Варикозная болезнь	1,42	1,01–2,03	<0,05
АГ	1,11	0,49–2,89	>0,05
ИБС	1,07	0,76–1,52	>0,05
ФП	0,94	0,60–1,46	>0,05
ХСН	1,16	0,80–1,68	>0,05
ОНМК+ТИА	1,09	0,66–1,80	>0,05
СД 2 типа	1,02	0,68–1,52	>0,05
Ожирение	0,86	0,60–1,22	>0,05
Болезнь Альцгеймера	4,91	0,89–27,04	>0,05
ХОБЛ	1,12	0,68–1,84	>0,05
Мочекаменная болезнь	1,07	0,66–1,73	>0,05
Язвенная болезнь желудка и двенадцатиперстной кишки	1,15	0,73–1,81	>0,05
Подагра	1,09	0,49–2,44	>0,05
Доброкачественная гиперплазия предстательной железы	2,03	0,71–5,79	>0,05

**Table:** Таблица 4. Распространенность и структура гериатрических синдромов

	С ОП (n=178, 29,3%)	%	Без ОП(n=429, 70,7%)	%	р
Количество гериатрических синдромов	6,83±3,0		5,52±2,64		0,028
Старческая астения	50	28,1	70	16,3	<0,001
Недостаточность питания	7	3,9	8	1,8	<0,001
Хроническая боль	82	46,1	184	42,9	0,392
Падения	93	52,2	217	50,6	0,355
Депрессия	20	11,2	85	19,8	0,028
Когнитивные нарушения (шкала MMSE, баллы)	27,2±4,1		27,8±4,1		0,239
Нарушения сна	85	47,8	160	37,3	0,324
Нарушения зрения	105	58,9	185	43,1	0,286
Нарушения слуха	73	41	163	37,9	0,219
Полипрагмазия	44	24,7	91	21,2	0,043
Количество принимаемых препаратов	4,56±2,4		4,26±2,1		0,069
Недержание мочи	53	29,7	87	20,3	0,003
Ортостатическая гипотония	9	5,1	24	5,6	0,282
Дефицит витамина D	19	10,6	46	10,7	0,253
Одинокое проживание	41	23	71	16,6	0,009
Головокружение	35	19,6	70	16,3	0,048
Гиподинамия	103	57,9	187	43,6	<0,001
Нарушения походки	53	29,8	113	26,3	0,212
Нарушения равновесия	85	47,7	216	50,3	0,256
Использование вспомогательных средств при передвижении	64	36	110	25,6	0,006

Согласно полученным данным, количество гериатрических синдромов, диагностированных у одного пациента, было выше у пациентов 1 группы. У пациентов с ОП достоверно чаще встречались такие гериатрические синдромы, как СА, гиподинамия и недостаточность питания, а также полипрагмазия, недержание мочи, головокружение. Пациенты с ОП чаще проживали одиноко и использовали вспомогательные средства при передвижении в сравнении с пациентами без ОП. При помощи однофакторного анализа была изучена взаимосвязь между ОП и гериатрическими синдромами (табл. 5).

Проведенный анализ продемонстрировал, что ОП ассоциирован (ОШ от 1.54 до 2.00) с такими гериатрическими синдромами, как СА, гиподинамия, использование вспомогательных средств при передвижении, нарушение сна, сенсорный дефицит по зрению, недержание мочи.

Отдельный интерес представляет ассоциация тяжелого ОП с наличием низкоэнергетических переломов и гериатрических синдромов (табл. 6).

Полученные данные показали, что тяжелый ОП с наличием низкоэнергетических переломов ассоциирован с другими гериатрическими синдромами: ортопедическая патология стопы, депрессия, тревога, хроническая боль. Связь ОП со СА была выявлена вне зависимости от тяжести ОП.

В рамках КГО также оценен функциональный статус пациентов, результаты представлены в табл. 7.

**Table:** Таблица 5. Ассоциации между остеопорозом и гериатрическими синдромами

	ОШ	95% ДИ	р
Старческая астения	2,00	1,32–3,04	<0,05
Нарушения зрения	1,54	1,08–2,19	<0,05
Гиподинамия	1,77	1,25–2,53	<0,05
Нарушения сна	1,54	1,08–2,29	<0,05
Использование вспомогательных средств при передвижении	1,63	1,12–2,37	<0,05
Недержание мочи	1,67	1,12–2,48	<0,05

**Table:** Таблица 6. Ассоциации между тяжелым остеопорозом и гериатрическими синдромами

	ОШ	95% ДИ	р
Ортопедическая патология стопы	4,42	2,54–7,68	<0,05
Депрессия	2,09	1,29–3,35	<0,05
Тревога	1,78	1,12–2,83	<0,05
Старческая астения	1,73	1,09–2,74	<0,05
Хроническая боль	1,10	1,06–1,19	<0,05

**Table:** Таблица 7. Оценка функционального статуса

	С ОП (n=178)	Без ОП (n=429)	р
«Возраст не помеха», средний балл	3,5±1,58	3,0±1,57	<0,001
Индекс Бартела, средний балл	88,5±14,1	93,4±9,7	0,002
Индекс Лоутона, средний балл	6,6±1,8	7,17±1,56	0,002
Скорость ходьбы, м/с	0,58±0,37	0,76±0,34	0,011
Время выполнения теста «Встань и иди», с	11,6±6,7	11,3±8,5	0,382
Суммарный балл за выполнение тандемных тестов	2,62±1,5	2,9±1,7	0,181
Суммарный балл по краткой батарее тестов физического функционирования	7,4±3,3	7,19±3,2	0,228
Пациенты со снижением мышечной силы рук, n (%)	33 (18,5)	131 (30,5)	0,331
Время, необходимое для 5 подъемов со стула, с	16,5±10,3	13,5±9,5	0,003

При этом оказалось, что пациенты 1 группы были более хрупкими, чаще имели высокий (5 и более) балл по шкале «Возраст не помеха», зависимость в базовой и инструментальной активности в повседневной жизни, а также низкую скорость ходьбы по сравнению с пациентами 2 группы. Необходимо отметить, что мы не получили достоверных различий в мышечной силе, однако в позиции мышечной функции наблюдались достоверные различия между пациентами обеих групп. Однако при проведении корреляционного анализа у пациентов с ОП выявлена слабая отрицательная корреляция между МПК и временем выполнения теста 5 подъемов со стула (r=-0,32; р=0,03), отражающего мышечную силу в ногах.

Нельзя не акцентировать внимание, что более половины обследуемых пациентов в течение последнего года перенесли падения. В связи с чем нам показалось целесообразным представить отдельно характеристику падений и их последствий (табл. 8).

**Table:** Таблица 8. Характеристика синдрома падений

	С ОП (n=178)	%	Без ОП (n=429)	%	р
Количество пациентов с падениями	93	52,2	217	50,6	0,304
Риск падений по шкале Морсе, баллы	33,1±18,7		33,7±20,2		0,158
Количество пациентов с высоким риском падений по шкале Морсе	36	20,2	80	18,6	0,253
Риск падений по шкале самооценки риска падений, баллы	5,87±3,65		5,63±3,3		0,226
Количество пациентов с высоким риском падений по шкале самооценки риска падений	124	69,7	301	70,2	0,859
Количество падений за последний год	2,2±1,7		2,1±1,9		0,343
Падения в анамнезе	83	89,2	161	74,2	0,009
Страх падений	40	43	106	48,8	0,298
Падения дома	32	34,4	64	29,5	0,292
Исход падений: перелом	14	15,1	17	7,8	0,040

Из таблицы 8 видно, что у пациентов с ОП в анамнезе чаще имелись указания на падения, а риск повторных падений был высоким. У пациентов 1 группы падения дома встречались несколько чаще. Важно отметить, что у пациентов 1 группы падения завершились переломами в 2 раза чаще, чем во 2 группе. Далее мы проанализировали все случившиеся после 60 лет переломы у пациентов с ОП. Оказалось, что 75 (42,1%) пациентов с ОП имели низкоэнергетические переломы. Из них 13 (17,3%) человек — множественные. Наиболее частыми локализациями случившихся переломов были: лучевая кость в типичном месте (29,3%), тела позвонков (14,7%), проксимальный отдел бедренной кости (24%).

## ОБСУЖДЕНИЕ

Репрезентативность выборок

Набор участников исследования проводился только в федеральном научном центре — РГНКЦ. В исследование вошли госпитализированные в гериатрическое отделение пациенты.

Сопоставление с другими публикациями

Распространенность ОП у пациентов, включенных в исследование, составила 29,3%, что соответствует эпидемиологическим данным в разных странах мира, в том числе в России и США [2][28–30]. Целью нашей работы было оценить коморбидный и гериатрический статус пациентов пожилого и старческого возраста с ОП и без него. Как оказалось, у пациентов 1 группы чаще наблюдались заболевания щитовидной железы, болезнь Альцгеймера, болезнь Паркинсона, анемия, остеоартрит, варикозная болезнь. Эти данные имеют подтверждение в литературе. Так, ряд авторов показали в наблюдательных исследованиях и метаанализах, что пациенты с болезнью Паркинсона имеют более высокий риск ОП и низкую МПК по сравнению с контролем. При этом оказалось, что тяжесть ОП коррелирует с тяжестью болезни Паркинсона [31][32]. Это объясняется тем, что дофаминовые рецепторы экспрессируются в остеобластах и остеокластах и влияют на гомеостаз костей [33]. Связь ОП и болезни Альцгеймера также показана в нескольких исследованиях. Оба заболевания являются многофакторными, включая генетические, метаболические, эндокринные и экологические. Частота переломов у пациентов с болезнью Альцгеймера более чем в три раза превышает частоту переломов у пациентов группы контроля с поправкой на возраст и пол [34][35].

Связь заболеваний щитовидной железы и ОП известна, однако патогенетические механизмы еще обсуждаются. Известно, что хондроциты и остеобласты непосредственно реагируют на гормоны щитовидной железы. Остеокласты также чувствительны к изменениям функции щитовидной железы, однако остается неопределенным, являются ли остеокласты клетками-мишенями или влияние гормонов щитовидной железы на резорбцию кости является косвенным за счет первичного действия гормонов на другие клетки [36][37].

Эпидемиологические данные о распространенности остеоартрита у пациентов с ОП достаточно противоречивы. Ряд авторов демонстрируют, что частота ОП обратно коррелирует с частотой остеоартрита, и эти два заболевания редко сосуществуют совместно у одного человека [38][39]. Однако исследования последних лет указывают на обратные данные и демонстрируют потерю костной ткани у пациентов с остеоартритом, даже на ранних стадиях заболевания. Достижения в области остеоиммунологии обнаружили влияние цитокинов на кость и обеспечили понимание роли провоспалительных цитокинов и адипокинов в метаболизме хряща и кости [40–42]. Полученные нами данные демонстрируют достоверное увеличение частоты остеоартрита у пациентов с ОП, а также наличие ассоциации между этими заболеваниями (ОШ 4,02; 95% ДИ 2,65–6,11; р<0,05).

Необходимо отметить, что большинство заболеваний, которые встречались достоверно чаще у пациентов 1 группы, являются возраст-ассоциированными. Единого механизма, объясняющего их взаимосвязь, на сегодняшний день нет. Наиболее удовлетворяющей является теория хронического системного воспаления. Данная концепция позволяет объяснить более частую встречаемость не только соматических заболеваний, но и гериатрических синдромов у пациентов с ОП. У пациентов 1 группы достоверно чаще встречались СА, недостаточность питания и гиподинамия (р<0,001). Эти синдромы определяют хрупкость пожилого человека. С другой стороны, возможно и обратное влияние. Многочисленные исследования и метаанализы демонстрируют повышенную секрецию ИЛ-1, ИЛ-6, ФНО при большинстве гериатрических синдромов: СА, саркопении, депрессии, болезни Альцгеймера, хроническом болевом синдроме [43–47]. Повышение провоспалительных цитокинов может обусловливать прогрессию ОП у гериатрических пациентов. Наши данные показывают наличие ассоциации между ОП и СА, гиподинамией, нарушением сна, недержанием мочи.

Клиническая значимость результатов

Полученные данные демонстрируют, что пациенты с ОП имеют большее число гериатрических синдромов, худший функциональный статус, достоверную связь с возраст-ассоциированными заболеваниями, чаще имеют инвалидность. В связи с чем наличие ОП, даже без ОП переломов, можно предложить рассматривать как маркер хрупкости. Тем более что самая сильная ассоциация ОП выявлена с синдромом СА (ОШ 2,00; 95% ДИ 1,32–3,04; р<0,05).

Ограничения исследования

Исследование проводилось у пациентов, госпитализированных в гериатрическое отделение стационара. Это выборка пациентов, которые заведомо тяжелее как по соматическому, так и гериатрическому статусу, чем пациенты, наблюдающиеся амбулаторно.

Направления дальнейших исследований

Считаем целесообразным проведение дальнейших исследований по изучению ассоциации ОП с гериатрическими синдромами, влияния не только на функциональный, но и когнитивный статус пожилых пациентов. Это позволит персонифицированно подходить к определению программы ведения пациентов пожилого и старческого возраста.

## ЗАКЛЮЧЕНИЕ

Пациенты с ОП представляют группу хрупких пациентов с отягощенным соматическим и гериатрическим статусом, что требует особого подхода к их ведению: обязательного проведения комплексной гериатрической оценки, по результатам которой определяется индивидуальный план ведения пожилого пациента.
